# DKK2 Impairs Tumor Immunity Infiltration and Correlates with Poor Prognosis in Pancreatic Ductal Adenocarcinoma

**DOI:** 10.1155/2019/8656282

**Published:** 2019-09-08

**Authors:** Jianyu Yang, Yongsheng Jiang, Ruizhe He, Wei Liu, Minwei Yang, Lingye Tao, Xueliang Fu, Yang Shen, Jiao Li, Dejun Liu, Yanmiao Huo, Junfeng Zhang, Rong Hua, Zhigang Zhang, Yongwei Sun

**Affiliations:** ^1^Department of Biliary-Pancreatic Surgery, Renji Hospital, School of Medicine, Shanghai Jiao Tong University, Shanghai, China; ^2^Department of Hepatobiliary Pancreas Surgery, Shanghai East Hospital, Tong Ji University School of Medicine, Shanghai, China; ^3^State Key Laboratory of Oncogenes and Related Genes, Shanghai Cancer Institute, Renji Hospital, School of Medicine, Shanghai Jiao Tong University, Shanghai, China

## Abstract

Pancreatic ductal adenocarcinoma (PDAC) remains one of the most devastating cancer types despite the improvement of modern medicine. In our present study, we found that dickkopf-related protein 2 (DKK2) shares a higher expression in PDAC compared with adjacent pancreas tissue in tissue microarray. In addition, an elevated expression of DKK2 predicts poorer prognosis of patients and positively correlated with poor tumor differentiation. Multivariate Cox regression analysis was also performed and confirmed that the expression of DKK2 is an independent prognostic factor in PDAC. A high expression of DKK2 correlates with cell migration and epithelial mesenchymal transition based on gene set enrichment analysis (GSEA) while knockdown of DKK2 in PDAC cells resulted in impaired cellular migration. Furthermore, GSEA predicts negative correlation between tumor immunity invasion and DKK2 expression. We then confirmed these results and demonstrated that a higher expression of DKK2 imparts the recruitment of CD8^+^ T cells. Our work suggested that DKK2 imparts tumor immune evasion and is associated with poor prognosis in pancreatic ductal adenocarcinoma.

## 1. Introduction

Pancreatic ductal adenocarcinoma (PDAC) is the sixth leading cause of cancer-related deaths in China and the fourth in Western countries [1, 2]. The mortality rates of PDAC are very close to its estimated incidence [3]. Although the death rates of most cancer have decreased due to improvements in early detection and treatment, the overall 5-year survival of pancreatic ductal adenocarcinoma patients has increased only slightly from 3% to 5%, on account of the early and aggressive local invasion and metastatic potential [4]. Surgery is the most effective curative option for PDAC patients, but only 20% are suitable for resection, and only around 20-25% of those survive to 5 years [5, 6]. Therefore, a hotspot in studying PDAC is identification of more prognostic indicators and biological markers. Some clinical and pathological factors, such as perineural invasion (PNI), lymph node metastasis, tumor stage, tumor grade, and tumor marker levels [7–9], extensively affected prognosis of PDAC. In addition, the prognostic values of altered genes/proteins involved in tumorigenesis and progression of PDAC, such as P53, CDKN2A, and DPC4 [10], were more and more explored.

Dickkopf 2 (DKK2), also known as secreted proteins with two cysteine-rich domains separated by a linking region, is a member of the dickkopf family which has been shown to be important in Wnt signaling regulation [11]. DKK2 is generally thought to be a direct inhibitor of Wnt binding to the LRP5/LRP6 coreceptors of FZD. More recently, DKK2 has been identified as a novel oncogene in various cancer types. Overexpression of DKK2 has been reported in Ewing sarcoma [12] and colorectal cancer [13]. But in melanoma, gastric cancer, renal cancer, and ovarian carcinoma, DKK2 expression is notably reduced [14–17]. Zhu et al. [17] demonstrated the relationship between epigenetic silencing of DKK2 and tumor growth, migration, and invasion in ovarian cancer. Also, they found that promoter hypermethylation of DKK2 might be a biomarker for ovarian cancer screening. Moreover, DKK2 played an important role in mediating osteolysis, invasiveness, and metastatic spread in Ewing sarcoma [12]. Recently, DKK2 was reported as a key player in stem cell signaling networks due to its function as a Notch signaling target [18]. Those investigations have revealed some potential links of DKK2 with cellular function to tumorigenesis which is very important for us to further understand how this protein contributes to cancer pathology.

However, the relation and prognostic significance between DKK2 and PDAC are unknown until now. Therefore, we examined DKK2 expression in 208 cases of formalin-fixed paraffin-embedded (FFPE) tissue specimens of human PDAC, performed univariate and multivariate analyses to correlate its expression levels with patient survival and clinicopathologic features in PDAC, and further explored the potential mechanism of DKK2 leading to poor prognosis in PDAC.

## 2. Materials and Methods

### 2.1. Ethics Statement

This research was approved by the ethics committee of Renji Hospital, Shanghai Jiao Tong University School of Medicine, for the use of samples. Informed consents were obtained from all patients before study inclusion.

### 2.2. Cell Lines and Transfection

Human PDAC cell lines AsPC-1 and CAPAN-1 were all purchased from the Cell Bank of the Chinese Academy of Sciences, and normal human pancreatic duct cell line hTERT-HPNE was purchased from American Type Culture Collection. All of these cells were cultured in specific medium supplemented with 10% (*v*/*v*) fetal bovine serum (FBS) and 1% antibiotics at 37°C in a humidified incubator under 5% CO_2_ condition. To interfere the expression of DKK2, the RNAiMAX reagent (Thermo Fisher, USA) was mixed with small interfering RNA targeting DKK2 and added to the culture medium.

### 2.3. Patients and PDAC Tissue Samples

Archived and formalin-fixed paraffin-embedded (FFPE) tissue samples were obtained from 208 patients diagnosed with PDAC, who had undergone surgical resection between January 2002 and June 2014 in the Biliary-Pancreatic Surgery Department of Renji Hospital, School of Medicine, Shanghai Jiao Tong University. All of the patients received ultrasound and computed tomography scans prior to surgery. None of these patients received radiotherapy, chemotherapy, or other anticancer therapies prior to surgery. Both tumor and no-tumor tissue samples were confirmed by histological proof. The clinicopathologic variables of the patients are summarized in Table 1. The stage was updated according to the current American Joint Committee on Cancer (AJCC) guidelines. Overall survival was measured from time of definitive operation to death from pancreatic cancer.

### 2.4. Tissue Microarray Construction

Tissue microarrays were constructed by Suzhou Xinxin Biotechnology Co. Ltd. (Xinxin Biotechnology Co., Suzhou, China). Tissue paraffin blocks of PDAC samples from 208 cases were stained with hematoxylin-eosin to confirm the diagnoses and marked at fixed points with most typical histological characteristics under a microscope. Two 1.5 mm cores per donor block were transferred into a recipient block tissue microarrayer, and each dot array contained fewer than 150 dots. Four-micrometer-thick sections were cut from the recipient block and transferred to glass slides with an adhesive tape transfer system for ultraviolet cross linkage.

### 2.5. Extraction of Total RNA and Quantitative Real-Time PCR

Total RNA from the primary tumor and adjacent nontumor tissue samples were extracted using TRIzol reagent (Invitrogen; Carlsbad, CA, USA) according to the manufacturer's instructions. The RNA was pretreated with RNase-free DNase, and 2 *μ*g RNA from each sample was used for cDNA synthesis. Quantitative real-time PCR was performed with SYBR Premix Ex Taq (Takara, Japan) on a 7500 Real-time PCR system (Applied Biosystems Inc., USA). Primer sequences used for DKK2 detection were as follows: forward: 5′-GATGATCCTTGGTGGGGAC-3′, reverse: 5′-GTACCAAGGACTGGCATTCG -3′. The relative expression of DKK2 was normalized to 18S RNA (forward: 5′-TGCGAGTACTCAACACCAACA-3′, reverse: 5′-GCATATCTTCGGCCCACA-3′). The PCR was run for 95°C for 2 min, followed by 40 cycles of 95°C for 10 s and 60°C for 31 s.

### 2.6. Immunohistochemical Staining

Detailed procedures were previously described [19] In brief, clinical tissues were fixed in paraffin. To stain, tissues were cut into sections which were then deparaffinized and rehydrated. 0.3% hydrogen peroxide and 10% BSA were used for antigen blockage separately. Primary antibody incubation lasts overnight at 4°C. Antibodies used in our work include DKK2 (21051-1-AP, Proteintech), CD8A (ab17147, Abcam), T-bet (sc-21763, Santa Cruz), GATA3 (ab199428, Abcam), and FOXP3 (22228-1-AP, Proteintech). Secondary antibody incubation lasts 1 hour in room temperature followed by diaminobenzidine tetrahydrochloride (DAB; Maixin Biotech, China) staining. The score of slice staining was based on both intensity and area. For intensity, no stain scored 0, weak stain scored 1, moderate stain scored 2, and strong stain scored 3. For area, 0-5% scored 0, 6%-35% scored 1, 36%-70% scored 2, and more than 70% scored 3. The final stain rate equals staining intensity multiplied by area: 0-1 rates “-,” 2-3 rates “+,” 4-6 rates “++,” and >6 rates “+++.”

### 2.7. Cell Migration Assay

Transwell and wound healing assays were performed to assess the migration capacity of tumor cells. Detailed procedures were previously described [20].

### 2.8. Western Blot (WB)

WB was conducted as reported earlier [21, 22]. Antibodies used were mouse anti-beta actin (1 : 5000, Abcam, ab8226), rabbit anti-DKK2 (1 : 1000, Proteintech, 21051-1-AP), E-cadherin (1 : 1000, Servicebio, GB14076), N-cadherin (1 : 1000, Servicebio, GB11135), CD8A (1 : 1000, ab17147, Abcam), and FOXP3 (1 : 1000, 22228-1-AP, Proteintech).

### 2.9. Chemotaxis Assay

To perform the chemotaxis assay, the healthy donor's peripheral blood mononuclear cells (PBMCs) were separated by the gradient centrifugation method using lymphocyte separation media (Biosera, France). Like the migration assay, PBMCs were seeded in the upper chamber and cultured for 6 hours. After that, the lower chamber medium was collected for counting. The cell number was determined by adding counting beads by flow cytometry. 10000 particles of beads were mixed with each group of medium containing migrating PBMC. The flow cytometry was performed by LSRFortessa (BD Biosciences, USA).

### 2.10. Data Mining and Statistical Analysis

Gene set enrichment analysis (GSEA) was performed on the Broad Institute platform. Samples are collected from The Cancer Genome Atlas (TCGA) and divided into two groups according to the expression level of DKK2. The false discovery rate (FDR) was set at 0.25.

Student's *t-*test was used to compare the DKK2 mRNA expression levels of the tumor tissue samples and the corresponding nontumor tissue samples. The chi-squared (*χ*^2^) test was used to analyze the correlations between DKK2 expression and clinicopathologic features in patients. Overall survival curves were calculated using the Kaplan-Meier method and were analyzed using the log-rank test. The Cox proportional hazards regression model was used to examine univariate and multivariate prognostic analyses. *P* value < 0.05 was considered to be statistically significant. All statistical analyses were performed with GraphPad Prism 5.0 software (San Diego, CA, USA) and SPSS 17.0 (SPSS, Chicago, IL, USA).

## 3. Results

### 3.1. Aberrantly Upregulated Expression of DKK2 in PDAC Both in mRNA and in Protein Levels

**Figure 1:**
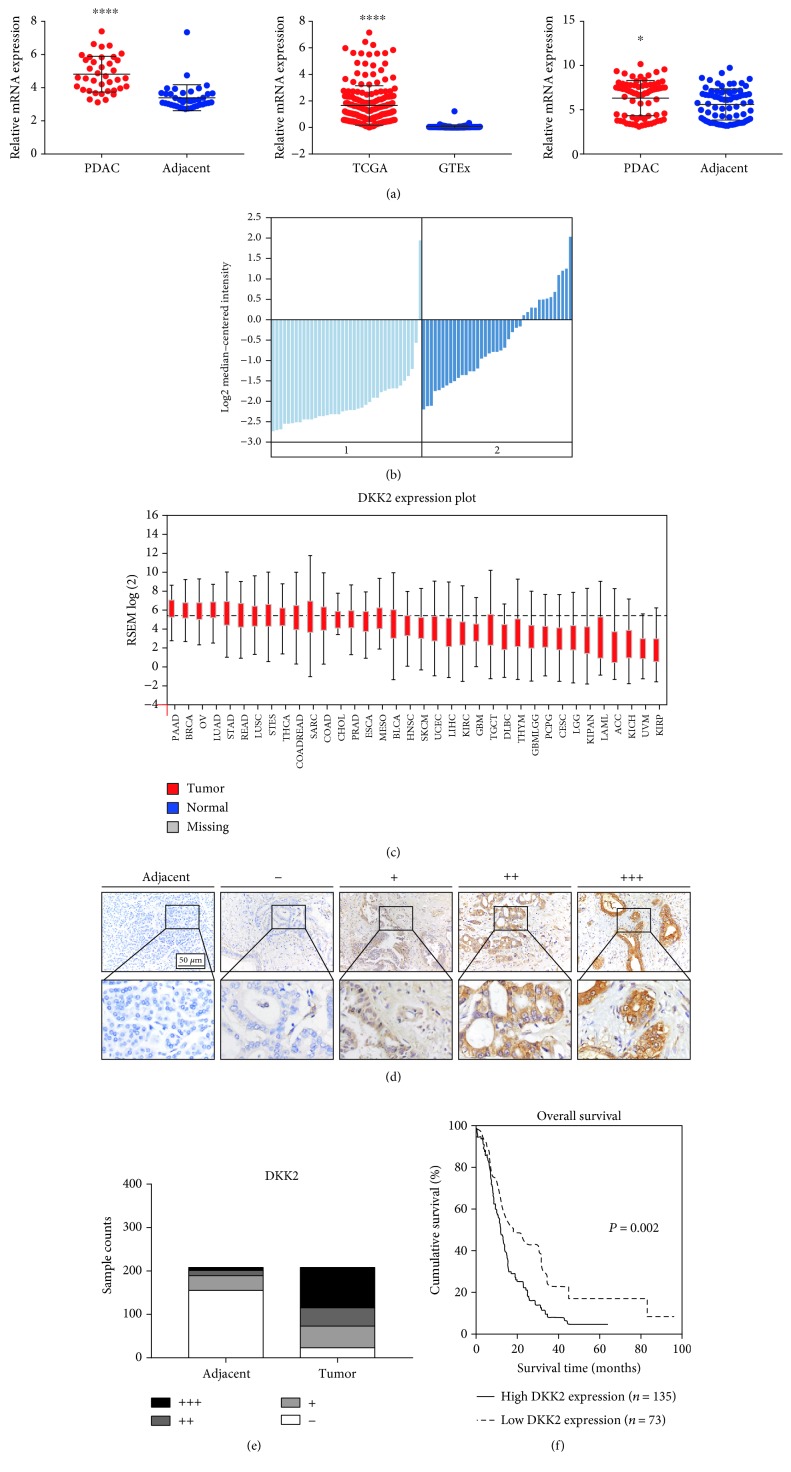
DKK2 was highly expressed in PDAC and was associated with poor prognosis in PDAC patients. (a, b) The mRNA expression level between tumor tissue and adjacent normal tissue in GSE15471 dataset, TCGA data, Oncomine, GTEx data, and Renji cohort. (c) DKK2 expression plot of various types of cancer from TCGA database. (d) Representative scoring criterion images of immunohistochemical staining of DKK2 expression in 208 paired PADC tumor and adjacent nontumor tissues. Scale bar: 50 *μ*m. (e) DKK2 was upregulated in PDAC tumor tissues. (f) High DKK2 expression correlated with inferior survival time. Abbreviations: TCGA: The Cancer Genome Atlas; GTEx: Genotype-Tissue Expression. Significant differences are indicated as ^∗^*P* < 0.05; ^∗∗∗∗^*P* < 0.0001.

To investigate the role of DKK2 in human pancreatic ductal adenocarcinoma, we first compared the mRNA expression level between tumor tissue and adjacent normal tissue in GEO database GSE15471, the Oncomine Badea pancreas data, The Cancer Genome Atlas (TCGA) data, and Genotype-Tissue Expression (GTEx) data. The result was verified by 50 pairs of PDAC and adjacent nontumor sample from the Renji cohort (Figures 1(a) and 1(b)). In addition, the expression of DKK2 in PDAC is the highest among almost all kinds of cancer types based on TCGA data (Figure 1(c)). We found that the mRNA expression level of DKK2 in tumor tissue is evidently higher than that in adjacent tissue especially in PDAC, which suggested that DKK2 is remarkably upregulated in PDAC tissue.

Furthermore, to validate our results in the protein level, PDAC tissue microarrays containing a cohort of 208 paired PDAC tumor and adjacent tissues were applied to immunohistochemistry staining. As shown in Figure 1(d), DKK2 is specifically expressed in cytoplasm of ductal-like cells. The expression pattern of DKK2 was measured based on both staining intensity and area. We found that the expression level of DKK2 in PDAC is much higher than that in adjacent tissue as shown in Figure 1(e). The specific scoring criteria are elucidated in Materials and Methods.

### 3.2. Relationship between DKK2 and Clinical Parameters in PDAC Patients

To investigate the relationship between the expression of DKK2 and clinical parameters in PDAC patients, chi-squared analysis was performed in the Renji TMA cohort. Clinical parameters include age, gender, tumor location, size, tumor differentiation, T classification, N classification, AJCC stage, and liver metastasis. Vascular invasion and vital status are analyzed with the corresponding patient's DKK2 expression intensity. The results showed that the expression of DKK2 positively correlated with poor tumor differentiation while no significant association was observed with other clinical parameters (Table 1).

### 3.3. Higher Expression of DKK2 Predicts Poorer Prognosis of PDAC Patients

**Figure 2:**
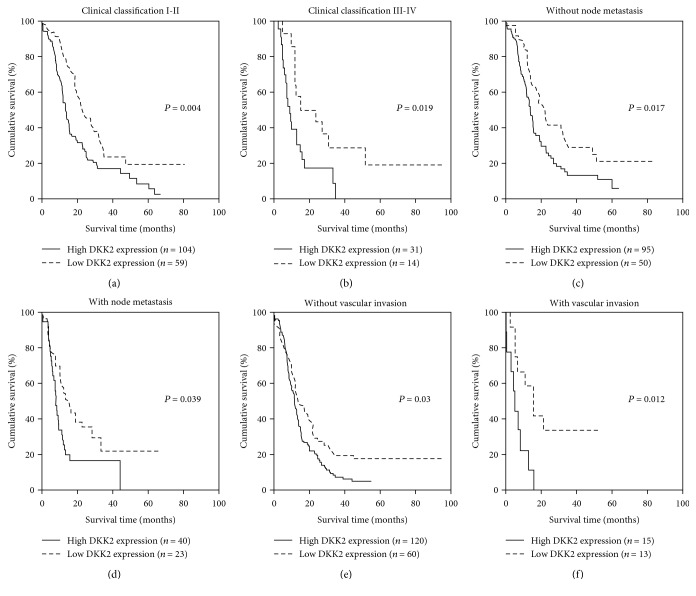
Prognostic significance of DKK2 assessed in PDAC patients classified by three conditions. The correlation between DKK2 expression and overall survival rate with patients grouped by clinical classification I-II stage (a), clinical classification III-IV stage (b), without node metastasis (c), with node metastasis (d), without vascular invasion (e), and with vascular invasion (f).

To evaluate the prognostic value of DKK2 expression in PDAC, Kaplan-Meier analysis and log-rank test were performed between DKK2 expression and the corresponding patient's follow-up information. As shown in Figure 1(f), patients with lower DKK2 expression possess longer survival time than those with higher DKK2 expression (*P* = 0.002). Also, we assessed the correlation between the DKK2 expression and the overall survival rate with patients grouped by clinic TNM stage classification, with or without lymph node metastasis and with or without vascular invasion. The results demonstrated that DKK2 is a prognostic marker representing shorter survival time despite the TNM stage, lymph node metastasis, and vascular invasion status (Figures 2(a)–2(f)).

In addition, we also performed univariate and multivariate analyses to determine the risk factors correlated with the patient's prognosis in TMA cohort. Univariate Cox regression analysis showed that DKK2 expression, tumor size, AJCC stage classification, and liver metastasis status are significantly associated with overall survival (Table 2). Furthermore, multivariate Cox regression analysis revealed that DKK2 expression and liver metastasis are risk factors for PDAC (Table 2). In conclusion, these data suggest that DKK2 presents prognostic value and might have effects on tumor progression.

### 3.4. DKK2 Acts as an Antagonist in the Wnt Pathway and Slightly Inhibits the Migration of Cancer Cells

**Figure 3:**
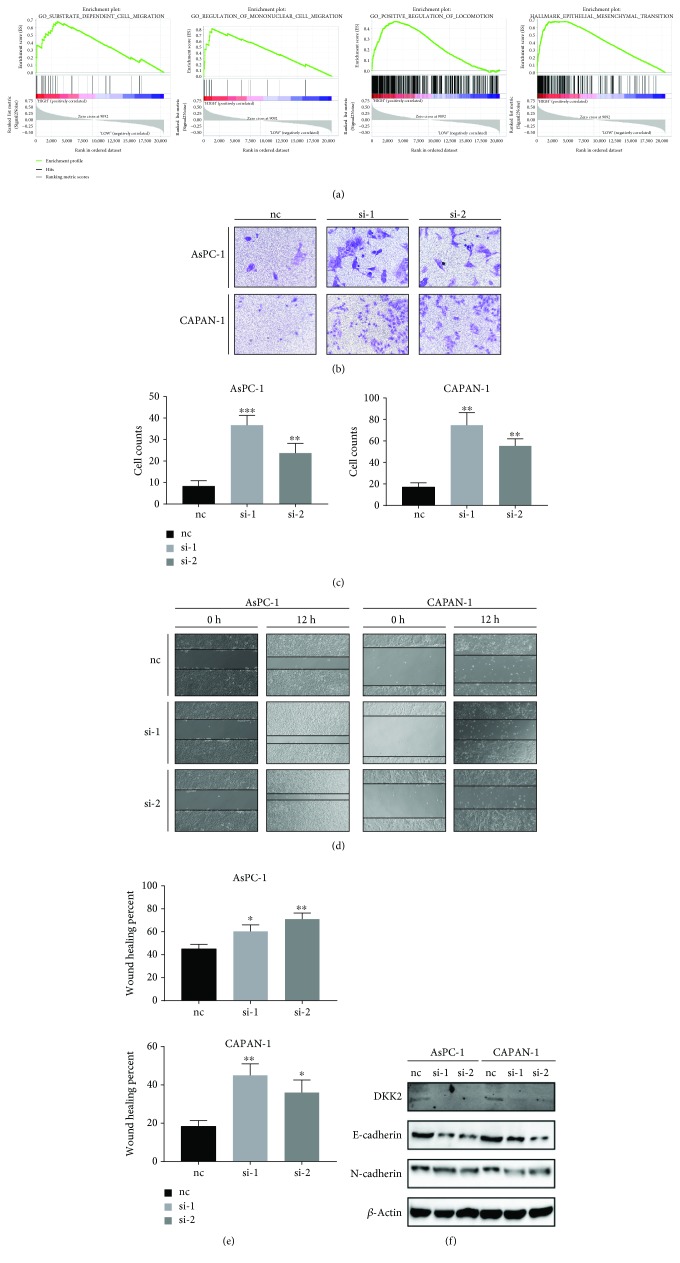
Knockdown of DKK2 promoted tumor cells' migration ability. (a) The enrichment plots in DKK2-highly expressed group compared to the low performed by gene set enrichment analysis. (b) Transwell assay showed that silencing DKK2 led to more migrated cells in AsPC-1 and CAPAN-1. For each transwell assay, the cell number was counted (c). (d) Movement of DKK2 knockout and control cells into the wound was shown in AsPC-1 and CAPAN-1 at 0 h and 12 h. The percentage of wound healing was assessed (e). Significant differences are indicated as ^∗^*P* < 0.05 and ^∗∗^*P* < 0.001.

Due to the negative relationship between the expression of DKK2 and clinical prognosis, we intended to explore the biological functions of DKK2 in tumor cells. As DKK2 is an important component in the Wnt pathway and gene set enrichment analysis (GSEA) also indicated that epithelial mesenchymal transition significantly enriched in the DKK-highly expressed group (Figure 3(a)), we first investigated whether DKK2 have an impact on the tumor cell's migration. The expression of DKK2 was interfered by small interfering RNA (siRNA) in two PDAC cell lines AsPC-1 and CAPAN-1. The transwell assay was performed in these two cell lines, and to our surprise, the result showed that with the expression of DKK2, fewer cells migrated to the downside of the chamber (Figures 3(b)–3(c)). This result was validated by the wound healing assay as cells with DKK2 interfered migrated to more areas than the negative control group as shown in Figures 3(d)–3(e). In Figure 3(f), the protein expression level of EMT genes in AsPC-1 and CAPAN-1 cell lines with DKK2 knockdown is examined using western blots which showed that DKK2 is negatively correlated with the expression of N-cadherin. These results indicated that the high expression of DKK2 in the tumor cell inhibits the migration capacity instead of enhances them.

### 3.5. The Expression of DKK2 Influences the Infiltration of Immune Cells in PDAC

**Figure 4:**
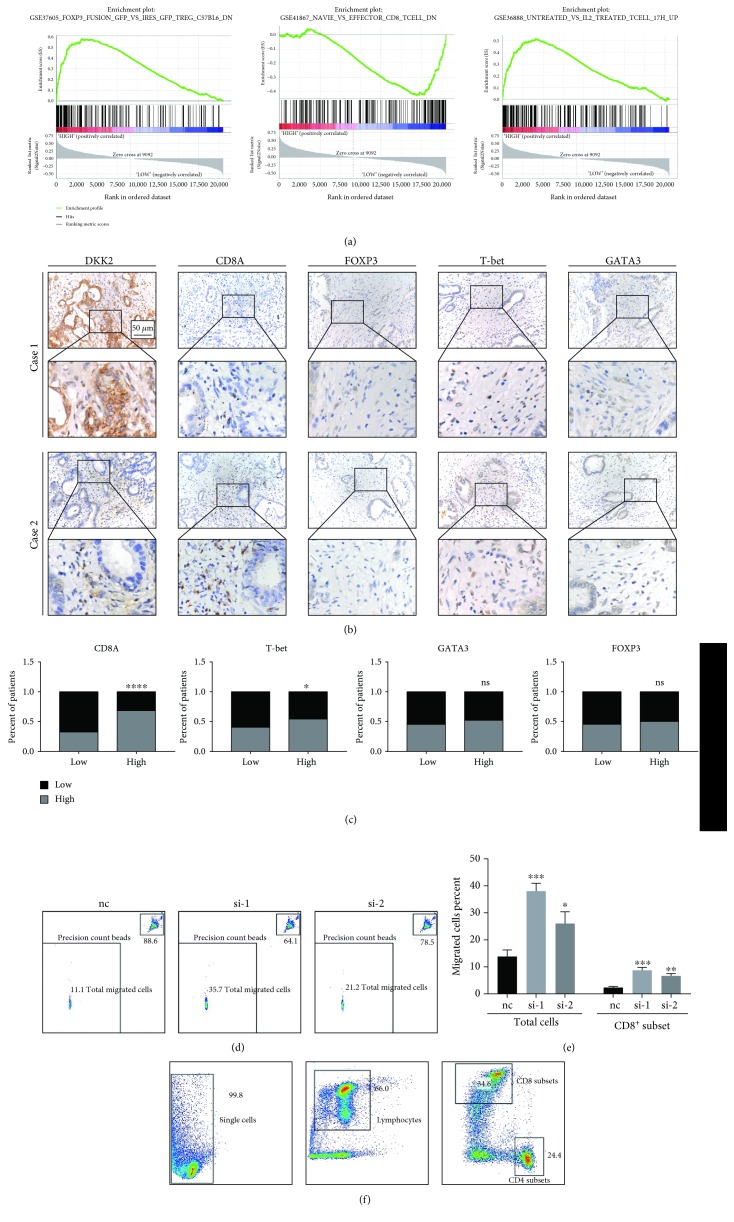
DKK2 impacted the infiltration capability of immune cells. (a) Gene set enrichment analysis of DKK2 showed the potential relationship between DKK2 and PDAC immunity invasion. (b) Representative immunohistochemical samples of DKK2, CD8A, FOXP3, GATA3, and T-bet in PDAC tissue microarray. (c) Patients were divided into two groups according to the expression of DKK2, and the column bar graphs showed the correlation between CD8A, FOXP3, GATA3, T-bet, and DKK2. (d) PBMCs treated with siRNA were performed with migration assay and then were counted by flow cytometry, and the percent was displayed by the bar graph (e). (f) Representative flow cytometry profiles of T cell subtype were shown. CD4^+^ and CD8^+^ cells, which were gated on single cells and lymphocytes, were gated as depicted. Significant differences are indicated as ^∗^*P* < 0.05, ^∗∗^*P* < 0.001, and ^∗∗∗^*P* < 0.0001.

Xiao and his colleagues found that the high expression of DKK2 in colorectal cancers (CRC) results in impaired tumor immune evasion [23]. We then sought to find out whether DKK2 plays the same role in PDAC. The result of GSEA suggests that DKK2 may participate in PDAC immunity invasion (Figure 4(a)). To confirm our hypothesis, we first performed IHC of different immune cells' markers including CD8A, FOXP3, GATA3, and T-bet in PDAC tissue microarray and calculated the correlation between DKK2 and each of them (Figures 4(b) and 4(c)). The results showed that patients with a higher expression of DKK2 companied with fewer infiltration of CD8 and Th1 cells. To further confirm the effect of DKK2 on immune cell recruitment, the chemotaxis assay was performed. Culture medium collected from separate groups of tumor cells was added in the lower chamber while peripheral blood mononuclear cells were seeded in the upper chamber. Flow cytometry was used to calculate the migrating cells. The results suggested that a higher concentration of DKK2 imparts the recruitment of total migrating cells (Figures 4(d) and 4(e)). We further studied the proportion of CD8^+^ T cells by flow cytometry (Figure 4(f)). The result suggested that DKK2 decreased the percentage of migrated CD8^+^ T cells significantly (Figure 4(e)). These results suggested that an aberrantly high expression of DKK2 leads to impaired recruitment of immune cells and decreased proportion of CD8^+^ T cells.

## 4. Discussion

In spite of the progression of modern medicine, pancreatic adenocarcinoma still remains one of the most fatal malignancies. With relatively low incidence, PDAC is the sixth leading cause of cancer-related deaths in China and the fourth in Western countries [1, 2]. Therefore, we determined to study the role of DKK2 in PDAC. As a member of the dickkopf family, the role of DKK2 varies depending on the cancer type. Though in most cases DKK2 acts as an antagonist of the Wnt signaling pathway, it is considered as an oncogene in a large portion of cancer types and so does is in our study. In our present work, we demonstrate that DKK2 may contribute to the progression of PDAC.

We first examined the expression pattern both in mRNA and in protein levels of DKK2 in PDAC and adjacent pancreas tissue. According to the published database and Renji cohort, the expression of DKK2 in PDAC is significantly higher than that of adjacent tissue. When analyzing the correlation between DKK2 and clinical parameters, we found that the expression of DKK2 positively correlated with tumor differentiation. Kaplan-Meier analysis indicates that a higher expression of DKK2 suggests poorer prognosis. Then, we performed univariable and multivariable Cox regression analyses which confirmed that DKK2 is an independent risk factor for poor prognosis of PDAC patients. Taken together, DKK2 may be an oncogene in PDAC.

Due to the negative relationship between DKK2 and clinical prognosis and participation in the Wnt pathway, we first want to determine whether DKK2 acts as an agonist of EMT in PDAC. Based on the results of GSEA, we found that a high expression of DKK2 does enrich in cell migration and EMT. But the cellular results suggested that knockdown of DKK2 did not impair cell migration, which is consistent with the antagonist role of DKK2 in the Wnt pathway reported by most researches. Thanks to the immunity-related pathway suggested by GSEA and Xiao and his colleagues' work that a high expression of DKK2 in colorectal cancers (CRC) results in impaired tumor immune evasion. We hypothesize that DKK2 in PDAC may damage immune cells' infiltration as well. We test our idea by comparing infiltrating condition of different immune cell types and the expression of DKK2 in TMA which indicated that the high expression of DKK2 correlates with fewer CD8^+^ T cells. The chemotaxis assay helped us determine that DKK2 do contribute to the poor infiltration of immune cells.

We all know that tumor cells could alter the composition or activities of immune cells that result in tumor-favorable microenvironment [24]. Our results suggest that DKK2 may be a tool secreted by tumor cells to modulate immune status. Higher composition of CD8^+^ cytotoxic T cells in PDAC associates with better prognosis while DKK2 leads to fewer CD8 counts suggested by our study [25, 26]. This may explain why a Wnt antagonist indicates poor prognosis. But we are not sure whether DKK2 directly bind the receptors on immune cells and function or act through other cell types such as tumor cells and pancreatic stellate cells (PSCs).

Our work first revealed that DKK2 was a novel predictive biomarker for poor prognosis in PDAC. We also investigate the impact of DKK2 on pancreatic cancer cells and status of immune cell infiltration. More work is needed to study how DKK2 function especially to determine its targeting cell type.

## Figures and Tables

**Table 1:** Correlations of DKK2 with clinical characteristics in PDAC patients.

Characteristics	Total	DKK2 expression		*P* value (*χ*^2^ test)
		High (*n* = 135)	Low (*n* = 73)	
Age (years)				0.55
<60	67	42	25	
≥60	141	93	48	
Gender				0.579
Male	120	76	44	
Female	88	59	29	
Tumor location				0.582
Head	139	92	47	
Body/tail	69	43	26	
Size				0.92
≤2 cm	25	16	9	
>2 cm	183	119	64	
Tumor differentiation				0.012
Well	11	11	0	
Moderate/poor	197	124	73	
T classification				0.876
T1	3	2	1	
T2	24	16	8	
T3	141	89	52	
T4	40	28	12	
N classification				0.779
Absent	145	95	50	
Present	63	40	23	
AJCC stage				0.88
Stage I	22	14	8	
Stage II	141	90	51	
Stage III	33	22	11	
Stage IV	12	9	3	
Liver metastasis				0.348
Absent	195	125	70	
Present	13	10	3	
Vascular invasion				0.177
Absent	180	120	60	
Present	28	15	13	
Vital status				0.229
Dead	180	114	66	
Alive	28	21	7	

**Table 2:** Univariate and multivariate analyses of prognostic parameters for survival in PDAC patients.

Prognostic parameter	Univariate analysis			Multivariate analysis		
	RR	95% CI	*P* value	RR	95% CI	*P* value
DKK2 (low vs. high)	1.729	1.24-2.412	**0.001** ^∗^	1.789	1.27-2.52	**0.001** ^∗^
Age (<60 vs. ≥60)	0.765	0.552-1.058	0.105			
Gender (male vs. female)	0.989	0.87-1.125	0.865			
Tumor location (head vs. body/tail)	0.999	0.713-1.399	0.996			
Size (≤2 cm vs. >2 cm)	1.729	1.030-2.902	**0.038** ^∗^	1.606	0.923-2.794	0.094
Tumor differentiation (well vs. moderate/poor)	2.125	0.938-4.813	0.071			
T classification (T1 vs. T2 vs. T3 vs. T4)	0.834	0.503-1.383	0.482			
AJCC stage (I vs. II vs. III vs. IV)	1.374	1.072-1.76	**0.012** ^∗^	0.927	0.678-1.266	0.633
N classification (absent vs. present)	0.788	0.317-1.959	0.608			
Liver metastasis (absent vs. present)	4.937	2.536-9.61	**<0.001** ^∗^	6.569	2.656-16.245	**<0.001** ^∗^
Vascular invasion (absent vs. present)	0.327	0.044-2.419	0.273			

Bold *P* value with asterisk means it has significant difference. RR: relative risk; CI: confidence interval; ^∗^*P* < 0.05.

## Data Availability

The data used to support the findings of this study are included within the article.

## Supplementary Materials

Supplementary Materials Supplementary Figure 1: (A) expression level of CD8A and FOXP3 in AsPC-1, CAPAN-1, and hTERT-HPNE cell lines using western blots.

## References

[1] Stathis A., Moore M. J. (2010). Advanced pancreatic carcinoma: current treatment and future challenges. *Nature Reviews Clinical Oncology*.

[2] Zhao P., Tian Y. T. (2010). Current status and the future of diagnosis and treatment of pancreatic cancer. *Zhonghua Zhong Liu Za Zhi*.

[3] Hidalgo M. (2010). Pancreatic cancer. *The New England Journal of Medicine*.

[4] Jemal A., Bray F., Center M. M., Ferlay J., Ward E., Forman D. (2011). Global cancer statistics. *CA: A Cancer Journal for Clinicians*.

[5] Butturini G., Stocken D. D., Wente M. N. (2008). Influence of resection margins and treatment on survival in patients with pancreatic cancer: meta-analysis of randomized controlled trials. *Archives of Surgery*.

[6] Neoptolemos J. P., Stocken D. D., Bassi C. (2010). Adjuvant chemotherapy with fluorouracil plus folinic acid vs gemcitabine following pancreatic cancer resection: a randomized controlled trial. *JAMA*.

[7] Lee S. R., Kim H. O., Son B. H., Yoo C. H., Shin J. H. (2013). Prognostic factors associated with long-term survival and recurrence in pancreatic adenocarcinoma. *Hepato-Gastroenterology*.

[8] Zhang J. F., Hua R., Sun Y. W. (2013). Influence of perineural invasion on survival and recurrence in patients with resected pancreatic cancer. *Asian Pacific Journal of Cancer Prevention*.

[9] Szkandera J., Stotz M., Absenger G. (2014). Validation of C-reactive protein levels as a prognostic indicator for survival in a large cohort of pancreatic cancer patients. *British Journal of Cancer*.

[10] Oshima M., Okano K., Muraki S. (2013). Immunohistochemically detected expression of 3 major genes (*CDKN2A/p16*, *TP53*, and *SMAD4/DPC4*) strongly predicts survival in patients with resectable pancreatic cancer. *Annals of Surgery*.

[11] Kawano Y., Kypta R. (2003). Secreted antagonists of the Wnt signalling pathway. *Journal of Cell Science*.

[12] Hauer K., Calzada-Wack J., Steiger K. (2013). DKK2 mediates osteolysis, invasiveness, and metastatic spread in Ewing sarcoma. *Cancer Research*.

[13] Matsui A., Yamaguchi T., Maekawa S. (2009). *DICKKOPF-4* and -*2* genes are upregulated in human colorectal cancer. *Cancer Science*.

[14] Kuphal S., Lodermeyer S., Bataille F., Schuierer M., Hoang B. H., Bosserhoff A. K. (2006). Expression of *Dickkopf* genes is strongly reduced in malignant melanoma. *Oncogene*.

[15] Maehata T., Taniguchi H., Yamamoto H. (2008). Transcriptional silencing of Dickkopf gene family by CpG island hypermethylation in human gastrointestinal cancer. *World Journal of Gastroenterology*.

[16] Hirata H., Hinoda Y., Nakajima K. (2009). Wnt antagonist gene DKK2 is epigenetically silenced and inhibits renal cancer progression through apoptotic and cell cycle pathways. *Clinical Cancer Research*.

[17] Zhu J., Zhang S., Gu L., di W. (2012). Epigenetic silencing of DKK2 and Wnt signal pathway components in human ovarian carcinoma. *Carcinogenesis*.

[18] Katoh M., Katoh M. (2007). WNT antagonist, DKK2, is a Notch signaling target in intestinal stem cells: augmentation of a negative regulation system for canonical WNT signaling pathway by the Notch-DKK2 signaling loop in primates. *International Journal of Molecular Medicine*.

[19] Jiang S. H., He P., Ma M. Z. (2014). PNMA1 promotes cell growth in human pancreatic ductal adenocarcinoma. *International Journal of Clinical and Experimental Pathology*.

[20] Wu D. J., Jiang Y. S., He R. Z. (2018). High expression of WNT7A predicts poor prognosis and promote tumor metastasis in pancreatic ductal adenocarcinoma. *Scientific Reports*.

[21] Jiang S. H., Li J., Dong F. Y. (2017). Increased serotonin signaling contributes to the Warburg effect in pancreatic tumor cells under metabolic stress and promotes growth of pancreatic tumors in mice. *Gastroenterology*.

[22] Hu L. P., Zhang X. X., Jiang S. H. (2019). Targeting purinergic receptor P2Y2 prevents the growth of pancreatic ductal adenocarcinoma by inhibiting cancer cell glycolysis. *Clinical Cancer Research*.

[23] Xiao Q., Wu J., Wang W. J. (2018). DKK2 imparts tumor immunity evasion through *β*-catenin-independent suppression of cytotoxic immune-cell activation. *Nature Medicine*.

[24] Pérez-Mancera P. A., Guerra C., Barbacid M., Tuvesonlow D. A. (2012). What we have learned about pancreatic cancer from mouse models. *Gastroenterology*.

[25] Fukunaga A., Miyamoto M., Cho Y. (2004). CD8+ tumor-infiltrating lymphocytes together with CD4+ tumor-infiltrating lymphocytes and dendritic cells improve the prognosis of patients with pancreatic adenocarcinoma. *Pancreas*.

[26] Degrate L., Nobili C., Franciosi C. (2009). Interleukin-2 immunotherapy action on innate immunity cells in peripheral blood and tumoral tissue of pancreatic adenocarcinoma patients. *Langenbeck's Archives of Surgery*.

