# Supplementary figures and images for: DKK2 Impairs Tumor Immunity Infiltration and Correlates with Poor Prognosis in Pancreatic Ductal Adenocarcinoma
Source: J Immunol Res. 2019 Sep 8;2019:8656282. doi: 10.1155/2019/8656282 (PMC6754880; doi:10.1155/2019/8656282)

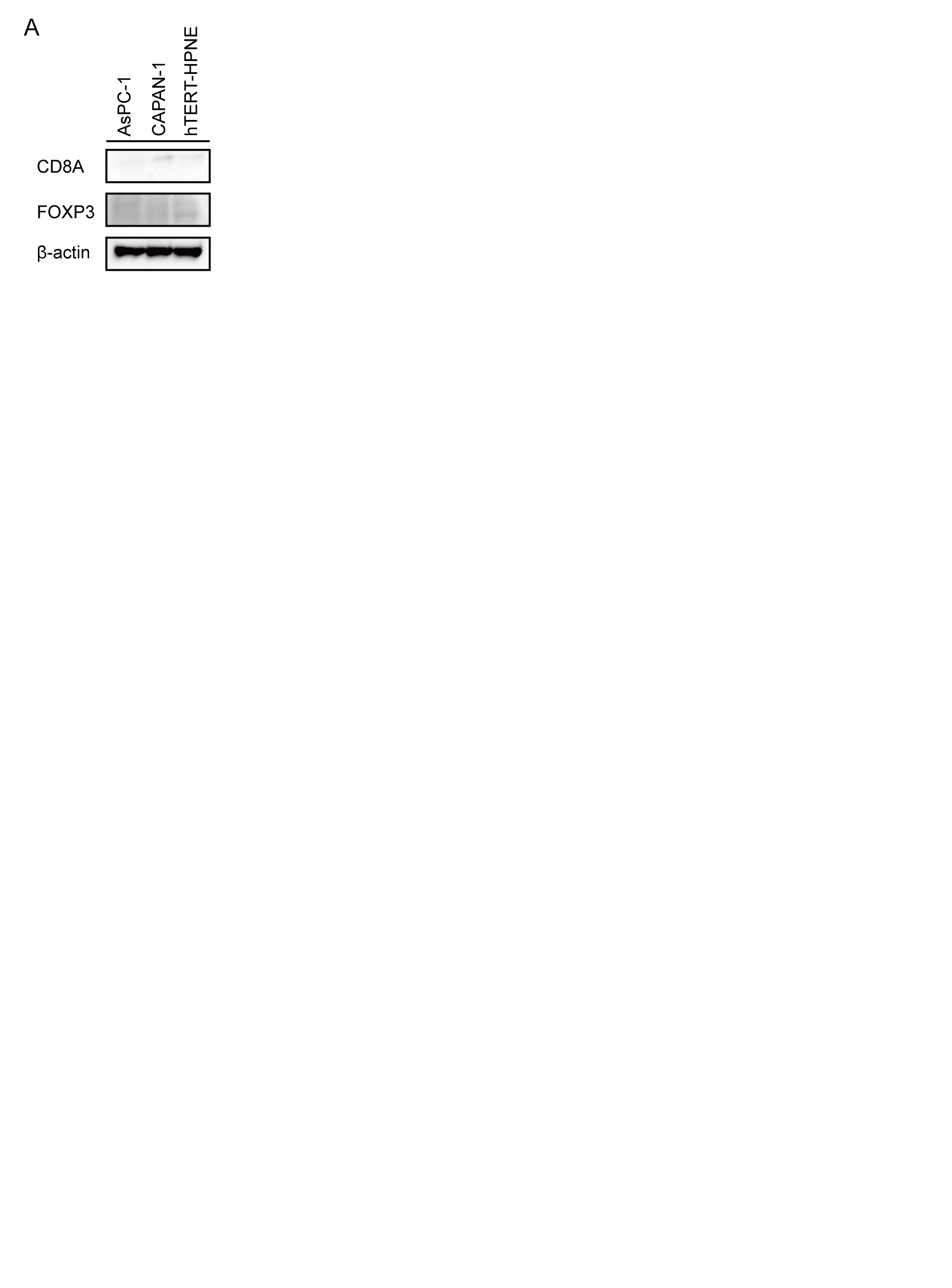

Supplement: Supplementary Materials — Supplementary Figure 1: (A) expression level of CD8A and FOXP3 in AsPC-1, CAPAN-1, and hTERT-HPNE cell lines using western blots. [file 8656282.f1.tif]
